# The association of body image distortion with weight control behaviors, diet behaviors, physical activity, sadness, and suicidal ideation among Korean high school students: a cross-sectional study

**DOI:** 10.1186/s12889-016-2703-z

**Published:** 2016-01-15

**Authors:** Jounghee Lee, Youngmin Lee

**Affiliations:** 1 Department of Nutrition Education, Graduate School of Education, Kyonggi University, Gyeonggido, 443-760 South Korea; 2 Department of Food and Nutrition, Seoul Women’s University, 621 Hwarangro, Nowongu, Seoul 139-774 South Korea

**Keywords:** Body image, Weight control, Older children, Physical activity, South Korea

## Abstract

**Background:**

The identification of predictors for body image distortion would be an especially important first step in targeting a vulnerable population and developing a nutrition intervention program. We aimed to investigate the prevalence of body image distortion and the factors associated with body image distortion among Korean high school students.

**Methods:**

We selected 20,264 normal weight high school students from the 10^th^ Korea Youth Risk Behavior Web-based Survey using nationally representative data in 2014. With multivariate logistic regression, we assessed the association of body image distortion with individual demographic and socio-economic factors, weight control behaviors, and mental health characteristics.

**Results:**

The over-estimation group of body weight status, compared with the correct estimation group, was significantly more likely to be a 3^rd^ year high school student [adjusted odds ratio (AOR): 1.27, 95 % confidence interval (CI): 1.16–1.39], to be female [AOR: 3.52, 95 % CI: 3.25–3.81], to employ unhealthy weight control behaviors [AOR: 1.54, 95 % CI: 1.37–1.72], and to have a sadness [AOR: 1.25, 95 % CI: 1.16–1.35] and suicidal ideation [AOR: 1.20, 95 % CI: 1.08–1.33]. The under-estimation group, compared with the correct estimation group, was significantly less likely to be female [AOR: 0.23, 95 % CI: 0.21–0.25] and to employ unhealthy weight control behaviors [AOR: 0.50, 95 % CI: 0.43–0.59] but were more likely to have a sadness [AOR: 1.12, 95 % CI: 1.03–1.21] and suicidal ideation [AOR: 1.12, 95 % CI: 1.00–1.25].

**Conclusions:**

Not only the over-estimation but also the under-estimation of body weight is prevalent among high school students in South Korea. Body image distortion was significantly associated with sadness and suicidal ideation.

**Electronic supplementary material:**

The online version of this article (doi:10.1186/s12889-016-2703-z) contains supplementary material, which is available to authorized users.

## Background

Misclassification of one’s weight status, including underweight as well as overweight status, especially among normal weight adolescents, has been a critical nutrition concern due to the associated negative physical health consequences such as unhealthy weight control behaviors and poor nutritional status [1, 2]. The self-perceived overweight adult dominantly practices unhealthy weight control methods including fasting, skipping meals, taking un-prescribed weight-loss pills and maintaining a one-food diet [3]. Unhealthy weight control behaviors are commonly characterized by extreme calorie restriction and nutrition imbalance that lead to short-term weight loss, increasing the risk to developing anemia, osteoporosis, and absent or irregular menstruation and dehydration [4]. Unhealthy weight control behaviors could be early warning signs of clinical eating disorders [5]. It is critical to identify this serious nutrition issue at an early stage.

There has been limited attention to body weight under-estimation, another type of weight misperception. A cross-sectional study (*n* = 5,443, South Korea) reported that male adolescents underestimated their weight status 2.7 times more than females, while female adolescents overestimated their weight status 2.3 times more than males [6]. Previous studies paid more attention to body weight overestimation among female adolescents or adults from school and college in South Korea, while information on body weight underestimation among male adolescents is scare [7, 8]. Therefore, we explored the prevalence and weight control behaviors of the body weight underestimation group using nationally representative data from Korean adolescents.

Previous studies revealed that body image distortion was significantly associated with feeling stressful and feeling depressed [7, 9, 10]. Weight misperception could be a valuable indicator of suicidal behaviors, such as suicidal ideation and suicidal attempts, among Korean adolescents [11]. In normal weight male adolescents, both self-perceived underweight and overweight males had significantly increased levels of depressive symptoms compared to subjects with accurate self-perceptions of their body weight in the United States [12]. Moreover, their elevated depressive symptoms persisted into early adulthood. This poor mental health status due to body weight perception lowers self-esteem, school achievement and the quality of life among adolescents [13–15].

The high prevalence of body image distortion calls for research to intervene in this critical nutrition issue. A recent study reported that approximately one out of two adolescents (aged 12–18) incorrectly perceived their body weight status, either underestimating (23.7 %) or overestimating (25.6 %) their body weight according to data from the 7^th^ Korea Youth Risk Behavior Web-based Survey in 2011 [16]. Due to its negative effects on both physical and mental health, it is imperative to assess the prevalence of body image distortion using the most up-to-date nationally representative data from South Korean adolescents.

Given the negative health consequences and the high prevalence of body image distortion, it is very important to identify the factors associated with body image distortion among high school students in South Korea. In the previous study, factors affecting misperception of body weight were limited to sociodemographic variables [16]. Therefore, using the 10^th^ Korea Youth Risk Behavior Web-based Survey (2014), which included a nationally representative sample of Korean adolescents, we examined the body weight perception of high school students. This study tested the hypothesis that demographic characteristics (year of high school and gender), subjective economic status, unhealthy weight control behaviors, feeling sad or despair, and suicidal ideation are associated with body image distortion. The aims of this study are to investigate (1) the prevalence of body image distortion; (2) weight control and dietary behaviors by body weight perception; and (3) the factors associated with body image distortion among Korean high school students.

## Methods

### Design

The 10^th^ Korea Youth Risk Behavior Web-based Survey is a self-report anonymous online survey using a nationally representative sample of Korean middle and high school students [17]. This survey was conducted by the Ministry of Education, Science and Technology, the Ministry of Health and Welfare, and the Korea Centers for Disease Control and Prevention in order to assess the health behavior of Korean adolescents (aged 12–18). The sample design of this survey included multistage sampling, clustering, and stratification. This survey used the 43-cluster sample strategy, with 129 strata identified, and the survey-sampling frame covered all of South Korea. This study was approved by the institutional review board of Seoul Women’s University, Seoul, South Korea.

### Participants

Survey sampling initially included 74,167 students registered in 400 middle schools and 400 high schools. Of these, 72,060 students participated in this survey, with a 97.2 % participation rate. We first selected high school students due to the age group of our interest (*n* = 35,904), and we excluded subjects who did not provide anthropometric data (*n* = 1,144). Finally, we selected 34,760 high school students for this analysis to examine the agreement between body image and actual body weight status. Then, we finally narrowed the sample to include only the normal weight subjects (*n* = 20,264) in this analysis.

### Measures

#### Demographic and socio-economic characteristics

We included demographic characteristics such as gender, age, and year in high school. We also assessed the education levels of their fathers and mothers using scales ranging from: 1) middle school or less, 2) high school, 3) college or more, and 4) unspecified. Subjective economic status was categorized into five groups: 1) high, 2) upper middle, 3) middle, 4) lower middle, and 5) low. The subjects’ school types were categorized into three groups: 1) co-ed high school, 2) men's high school, and 3) women’s high school. In Korea, there are single-gender high schools as well as co-ed high schools (mixed-gender high school).

#### Anthropometric measures

The survey questionnaire included the self-reported weight and height of the subjects. To calculate the body mass index (BMI) of subjects, the weight of the subjects in kilograms was divided by the height in meters squared (kg/m^2^). We classified the BMI of the subjects into 3 groups by age using the extended international BMI cut-offs for children [18]. If subjects were 18-year-old high school students, we employed the same BMI cut-offs used for adults: underweight (BMI < 18.50), normal weight (18.50 ≤ BMI < 23.00), and overweight or obese (BMI ≥ 23.00). For 15-17-year-old subjects, we used the specific BMI values corresponding to the Asian adult equivalent BMI of 23 for overweight and 25 for obese (Additional file 1: Table S1).

#### Subjective body image perception

We assessed subjective body image perception using the following question: “What do you think of your body image?”. Subjects checked one of the following responses: (1) very thin, (2) slightly thin, (3) normal, (4) slightly fat, and (5) very fat.

#### Self-reported weight control attempts and weight control behaviors

The survey questionnaire included the self-reported weight control attempts of the subjects in the last 30 days. To answer the weight control attempt questions, the subjects checked one of four options: 1) doing nothing to control weight, 2) making an effort to lose weight, 3) making an effort to gain weight, and 4) making an effort to maintain weight. Weight control behaviors consisted of 9 methods: 1) doing regular exercise, 2) fasting for ≥ 24 h, 3) eating less, 4) taking prescription weight loss medication, 5) taking non-prescription weight loss medication, 6) taking laxatives or diuretics, 7) vomiting, 8) eating a one-food diet, 9) taking Chinese medicine, and 10) eating food substitutes (e.g., powder or a special drink). To classify unhealthy weight control behaviors, we included the following: 1) fasting for ≥ 24 h, 2) taking non-prescription weight loss medication, 3) taking laxatives or diuretics, 4) vomiting, 5) eating a one-food diet, and 6) eating food substitutes [1]. We measured fasting by asking the subjects with the following question: “Have you had fasting (not eat anything) for ≥ 24 h during the past 30 days?” Subjects checked either one the following: 1) No, I have not had fasting; or 2) Yes, I have had fasting.

#### Dietary behaviors

The frequencies of eating breakfast, lunch and dinner in the last 7 days were recorded as continuous variables. The consumption of bread, and powder made of mixed grains, roasted and ground grains was considered a meal. However, drinking only milk or juice was not considered a meal. To assess dietary behaviors, we also included the frequency of eating foods such as vegetables (excluding ‘kimchi’, pickled cabbage), fruits (excluding fruit juice), milk, soda, and fast food (e.g., pizza, hamburger, and fried chicken) during the past 7 days. The responses to each question were classified into five groups: 1) none, 2) 1–2 times, 3) 3–4 times, 4) 5–6 times, and 5) once or more a day.

#### Physical activity

We examined the frequency of doing physical activity for 60 min or more per day in the last 7 days. Physical activity included all types of activity that was accompanied by an increased heart rate or fast breathing. We assessed the frequency of walking for 10 min or more in the last 7 days. Physical activity and walking were categorized into five groups: 1) none, 2) 1–2 days, 3) 3–4 days, 4) 5–6 days, and 5) daily. We also examined the frequency of vigorous exercise and muscle strengthening exercises in the last 7 days. Vigorous exercise and muscle strengthening exercise were classified into four groups: 1) none, 2) 1–2 days, 3) 3–4 days, and 4) 5 days or more. Vigorous exercise was accompanied by very fast breathing or sweating. Muscle strengthening exercises included push-ups, sit-ups, weight-lifting, and exercising on the horizontal bar or the parallel bar.

#### Sadness

We assessed sadness among the subjects with the question: “In the past year, have you ever felt sad or despaired so that your feelings disturbed everyday life for whole two weeks?” Subjects responded with the following: 1) No, I never felt it; or 2) Yes, I have felt it.

#### Suicidal ideation

We examined whether the subjects ever thought of suicide in the past year. The responses were recorded as follows: 1) No, I never thought of it; or 2) Yes, I have thought of it.

### Statistical analyses

We tested differences among the continuous variables (i.e., frequency of eating breakfast/ lunch/ dinner) using one-way ANOVA (Analysis of variance) and used Chi-square tests for the categorical data (i.e., weight control attempts and behaviors, dietary behaviors, physical activity). We examined agreement between subjective body image and actual body weight status based on BMI using cross-tabulation and Kappa statistics of agreement [19]. We compared the demographic and socio-economic characteristics with body image perception among normal weight high school students by using a multivariate logistic regression [20]. We also examined any differences in weight control attempts, weight control behaviors, dietary behaviors, and physical activity stratified by body image perception. Then, we utilized multivariate logistic regression to compare self-perceived underweight and overweight subjects with subjects that correctly estimated their own body weight status as the reference group. This study examined the following hypotheses: 1) self-reported weight control attempt, weight control behaviors, dietary behaviors, and physical activity are different by body image perception; 2) year of high school, gender, subjective economic status, unhealthy weight control behaviors, feeling sad or despair, and suicidal ideation are associated with body image misperception. To test our hypothesis, *P*-values < 0.05 were considered significant in all statistical analyses. We analyzed the data set using SPSS 21.0 (SPSS Inc., Chicago, IL).

## Results

### Demographic characteristics of the subjects

This study included 20,264 normal weight subjects, 47.7 % of males and 52.3 % of female high school students (Table 1). The mean age of the subjects was 16.43 years. Almost half of the subjects’ fathers had an educational achievement of a college diploma or more (48.9 %), while 44.6 % of the subjects’ mothers had an educational achievement of a high school diploma. Almost one of two subjects (50.4 %) perceived their socio-economic status as middle class followed by upper middle class (22.5 %) and lower middle class (17.6 %). In terms of the school type, 59.6 % of subjects went to co-ed high schools, 18.6 % went to men’s high schools, and 21.8 % went to women’s high schools.

**Table 1:** General characteristics of normal weight high school students

	Unweighted	Weighted	Weighted % ± SE
	(*N* = 20,264)	(*N* = 1,025,792)	
Gender			
Male	9,211	489,237	47.7 ± 1.9
Female	11,053	536,555	52.3 ± 1.9
Age (years)			16.43 ± 0.01^a^
Father’s education			
Middle school or less	710	34,766	3.4 ± 0.1
High school	6,970	349,989	34.1 ± 0.6
College or more	9,814	502,048	48.9 ± 0.8
Unspecified	1,903	96,159	9.4 ± 0.3
Not applicable	867	42,830	4.2 ± 0.1
Mother’s education			
Middle school or less	614	29,614	2.9 ± 0.1
High school	9,088	457,343	44.6 ± 0.6
College or more	7,875	401,145	39.1 ± 0.7
Unspecified	1,901	987,10	9.6 ± 0.3
Not applicable	786	38,977	3.8 ± 0.2
Socio-economic status			
High	999	51,065	5.0 ± 0.2
Upper middle	4,551	231,158	22.5 ± 0.4
Middle	10,247	517,446	50.4 ± 0.4
Lower middle	3,549	180,557	17.6 ± 0.3
Low	891	45,545	4.4 ± 0.2
Year of high school			
1st year	6,600	334,603	32.6 ± 0.3
2nd year	6,889	346,948	33.8 ± 0.3
3rd year	6,775	344,239	33.6 ± 0.3
School type			
Co-ed high school	12,460	611,734	59.6 ± 2.3
Men’s high school	3,524	190,439	18.6 ± 1.9
Women’s high school	4,280	223,619	21.8 ± 2.1

^a^ mean ± SE

### Body weight status and subjective body image

Overall, three out of five students correctly perceived their actual body weight status (58.8 % of males vs. 64.1 % of females). The Kappa statistics showed moderate agreement among all subjects (Kappa = 0.41); the agreement was fair in male subjects (Kappa = 0.39) and the female Kappa value was moderate (Kappa = 0.44) (Additional file 1: Table S2).

We also found that about a quarter (22.8 %) of subjects underestimated their actual body weight status. More male subjects underestimated their body weight status than female subjects (36.2 % of males vs. 9.5 % of females). On the other hand, 26.4 % of female subjects overestimated their body weight status compared to only 5.0 % of male subjects (Additional file 1: Table S2).

### Self-reported weight control attempts and weight control behaviors

A significantly higher percentage of subjects in the under-estimation group did nothing to control their weight (66.5 %) compared to 54.5 % in the correct estimation group and 40.3 % in the overestimation group (Table 2). A significantly higher percentage of subjects in the under-estimation group made an effort to gain weight (18.4 %), followed by the correct estimation group (2.7 %) and lastly the over-estimation group (0.4 %). Half of subjects in the over-estimation group made an effort to lose weight (49.6 %). However, almost one out of three subjects made an effort to lose weight in the correct estimation group (30.6 %).

**Table 2:** Self-reported weight control attempts and weight control behaviors by body image perception among normal weight high school students

	Under-estimation	Correct estimation	Over-estimation	Total	*P*-value
	(*n* = 5,990)	(*n* = 9,387)	(*n* = 4,887)	(*n* = 20,264)	
Self-reported weight control attempt during the past 30 days					
Doing nothing to control weight	66.5 ± 0.6	54.5 ± 0.7	40.3 ± 0.7	54.8 ± 0.6	<0.001
Making an effort to lose weight	9.1 ± 0.4	30.6 ± 0.8	49.6 ± 0.7	28.6 ± 0.7	
Making an effort to gain weight	18.4 ± 0.6	2.7 ± 0.2	0.4 ± 0.1	6.9 ± 0.3	
Making an effort to maintain weight	5.9 ± 0.3	12.1 ± 0.3	9.8 ± 0.4	9.7 ± 0.2	
Weight control behaviors	(*n* = 2,024)	(*n* = 4,309)	(*n* = 2,925)	(*n* = 9,258)	
Doing regular exercise	70.0 ± 0.9	69.3 ± 0.8	66.1 ± 0.9	68.5 ± 0.5	<0.01
Fasting (≥24 h)	5.4 ± 0.5	8.6 ± 0.5	10.4 ± 0.6	8.5 ± 0.3	<0.001
Eating less	29.1 ± 1.4	73.4 ± 1.0	88.0 ± 0.7	68.1 ± 1.1	<0.001
Taking prescription weight loss medication	1.3 ± 0.2	1.0 ± 0.2	1.6 ± 0.2	1.3 ± 0.1	0.085
Taking non-prescription weight loss medication	1.4 ± 0.2	1.5 ± 0.2	3.2 ± 0.3	2.0 ± 0.1	<0.001
Taking laxatives or diuretics	1.8 ± 0.3	2.1 ± 0.2	3.3 ± 0.3	2.4 ± 0.2	<0.01
Vomiting	2.0 ± 0.3	2.3 ± 0.2	3.5 ± 0.3	2.6 ± 0.2	<0.01
Eating one-food diet	2.9 ± 0.3	6.7 ± 0.4	9.5 ± 0.5	6.7 ± 0.3	<0.001
Taking Chinese medicine	7.8 ± 0.6	3.0 ± 0.3	3.1 ± 0.3	4.1 ± 0.2	<0.001
Eating food substitute	3.7 ± 0.4	8.0 ± 0.4	12.8 ± 0.7	8.5 ± 0.3	<0.001

Weighted % ± SE

Among subjects attempting to control their weight, the most common weight control behavior was ‘doing regular exercise’ (68.5 %), followed by ‘eating less’ (68.1 %), fasting ≥24 h (8.5 %), eating food substitute (8.5 %), and eating one-food diet (6.7 %). The most common type of weight control behavior was ‘doing regular exercise’ (70.0 %) in the under-estimation group, while it was ‘eating less’ in the other two groups (73.4 % in the correct-estimation group, and 88.0 % in the over-estimation group). After excluding the two common types of weight control behaviors (i.e., ‘doing regular exercise’ and ‘eating less’) in the three groups, the weight control behaviors were ‘taking Chinese medicine’ (7.8 %) and fasting for ≥24 h (5.4 %) in the under-estimation group; fasting for ≥24 h (8.6 %), ‘eating food substitute’ (8.0 %) and ‘eating one-food diet’ (6.7 %) in the correct estimation group; ‘eating food substitute’ (12.8 %), fasting for ≥24 h (10.4 %) and ‘eating one-food diet” (9.5 %) in the over-estimation group.

### Dietary behaviors

The breakfast-eating frequency was significantly higher in the under-estimation group (4.40 times) and the correct estimation group (4.39 times) compared to 4.27 times in the over-estimation group (Table 3). The lunch-eating frequency was not significantly different among the three groups. The dinner-eating frequency was significantly higher in the under-estimation group (5.98 times) followed by 5.86 times in the correct estimation group and 5.70 times in the over-estimation group (*P* < 0.001).

**Table 3:** Dietary behaviors by body image perception among normal weight high school students

	Under-estimation	Correct estimation	Over-estimation	Total	*P*-value
	(*n* = 5,990)	(*n* = 9,387)	(*n* = 4,887)	(*n* = 20,264)	
	mean ± SE				
Frequency of eating breakfast	4.40 ± 0.04^a^	4.39 ± 0.03^a^	4.27 ± 0.04^b^	4.36 ± 0.03	<0.01
Frequency of eating lunch	5.98 ± 0.03	6.03 ± 0.02	6.06 ± 0.03	6.02 ± 0.02	0.193
Frequency of eating dinner	5.98 ± 0.03^a^	5.86 ± 0.02^b^	5.70 ± 0.03^c^	5.86 ± 0.18	<0.001
	weighted % ± SE				
Frequency of eating vegetables during the past 7 days					
None	3.7 ± 0.2	3.3 ± 0.2	4.3 ± 0.3	3.7 ± 0.1	<0.05
1–2 times	16.7 ± 0.5	17.1 ± 0.4	17.7 ± 0.5	17.1 ± 0.3	
3–4 times	26.0 ± 0.6	24.4 ± 0.4	25.6 ± 0.6	25.2 ± 0.3	
5–6 times	14.2 ± 0.4	14.9 ± 0.4	14.0 ± 0.5	14.5 ± 0.3	
≥1 time per day	39.4 ± 0.6	40.2 ± 0.6	38.4 ± 0.7	39.6 ± 0.4	
Frequency of eating fruits during the past 7 days					
None	8.2 ± 0.4	8.0 ± 0.3	8.3 ± 0.4	8.1 ± 0.2	0.293
1–2 times	33.5 ± 0.7	31.6 ± 0.5	32.9 ± 0.7	32.5 ± 0.4	
3–4 times	29.4 ± 0.6	30.2 ± 0.5	29.6 ± 0.6	29.8 ± 0.3	
5–6 times	10.4 ± 0.4	11.1 ± 0.3	11.2 ± 0.4	10.9 ± 0.2	
≥1 time per day	18.6 ± 0.6	19.0 ± 0.5	18.0 ± 0.6	18.7 ± 0.4	
Frequency of milk consumption during the past 7 days					
None	17.5 ± 0.6	19.3 ± 0.5	23.7 ± 0.6	19.8 ± 0.4	<0.001
1–2 times	24.1 ± 0.6	26.8 ± 0.6	29.0 ± 0.7	26.5 ± 0.4	
3–4 times	21.3 ± 0.6	21.0 ± 0.4	20.5 ± 0.6	21.0 ± 0.3	
5–6 times	14.2 ± 0.6	12.4 ± 0.4	11.3 ± 0.5	12.7 ± 0.3	
≥1 time per day	23.0 ± 0.6	20.6 ± 0.6	15.5 ± 0.6	20.1 ± 0.5	
Frequency of soda consumption during the past 7 days					
None	21.0 ± 0.6	25.9 ± 0.6	28.6 ± 0.7	25.0 ± 0.5	<0.001
1–2 times	48.1 ± 0.6	49.5 ± 0.5	51.0 ± 0.7	49.4 ± 0.4	
3–4 times	20.7 ± 0.5	17.8 ± 0.5	14.7 ± 0.5	17.9 ± 0.4	
5–6 times	5.5 ± 0.3	3.7 ± 0.2	3.1 ± 0.3	4.1 ± 0.2	
≥1 time per day	4.7 ± 0.3	3.2 ± 0.2	2.5 ± 0.2	3.5 ± 0.2	
Frequency of fast-food consumption during the past 7 days					
None	20.8 ± 0.5	22.2 ± 0.4	22.3 ± 0.6	21.8 ± 0.3	<0.01
1–2 times	61.3 ± 0.6	61.5 ± 0.5	61.6 ± 0.7	61.5 ± 0.4	
3–4 times	13.9 ± 0.4	13.5 ± 0.4	13.5 ± 0.5	13.6 ± 0.3	
5–6 times	2.5 ± 0.2	1.8 ± 0.1	1.6 ± 0.2	2.0 ± 0.1	
≥1 time per day	1.5 ± 0.2	1.0 ± 0.1	1.0 ± 0.1	1.1 ± 0.1	

Different letters in superscript indicate significant differences in mean values

Less than half of all subjects (39.6 %) consumed vegetables one time or more per day during the past 7 days. Additionally, almost one out of five subjects consumed fruits one time or more per day during the past 7 days. The percentage of subjects who drank milk daily was lowest in the over-estimation group (15.5 %) compared to 23.0 % in the under-estimation group and 20.6 % in the correct estimation group. The majority (49.4 %) of subjects consumed soda one or two times during the past 7 days, and the percentage of daily soda consumption was significantly higher showing 4.7 % in the under-estimation group than 3.2 % in the correct-estimation group and 2.5 % in the over-estimation group. More than half of the subjects (61.5 %) consumed fast-food 1–2 times during the past 7 days, and about one out of five subjects (21.8 %) did not consume any fast food during the past 7 days.

### Physical activity

Less than 5 % of subjects did physical activity daily for 60 min or more during the past 7 days; the percentage of subjects who did daily physical activity for ≥ 60 min was lowest in the overestimation group (2.5 %) compared to 5.4 % in the under-estimation group and 4.9 % in the correct estimation group (Table 4). Almost 30 % of subjects did not do any vigorous exercise during the past 7 days. More than half of subjects did not do any muscle strengthening exercise during the past 7 days (51.0 %). The percentage of subjects that did muscle strengthening exercise 5 days or more was highest in the under-estimation group (15.2 %) compared to those in the correct estimation group (11.3 %) and the overestimation group (5.1 %). About half of the subjects walked for 10 min or more daily during the past 7 days.

**Table 4:** Physical activity by body image perception among normal weight high school students

	Under-estimation	Correct estimation	Over-estimation	Total	*P*-value
	(*n* = 5,990)	(*n* = 9,387)	(*n* = 4,887)	(*n* = 20,264)	
Doing physical activity for ≥ 60 min during the past 7 days					
None	30.5 ± 0.7	37.1 ± 0.7	45.1 ± 0.8	37.0 ± 0.6	<0.001
1–2 days	35.8 ± 0.7	35.3 ± 0.5	34.6 ± 0.7	35.3 ± 0.4	
3–4 days	20.1 ± 0.6	15.8 ± 0.4	12.9 ± 0.5	16.4 ± 0.4	
5–6 days	8.2 ± 0.4	6.9 ± 0.3	5.0 ± 0.3	6.8 ± 0.2	
Daily	5.4 ± 0.3	4.9 ± 0.3	2.5 ± 0.2	4.5 ± 0.2	
Doing vigorous exercise during the past 7 days					
None	21.4 ± 0.7	28.5 ± 0.7	36.1 ± 0.8	28.2 ± 0.6	<0.001
1–2 days	42.4 ± 0.7	41.2 ± 0.5	42.7 ± 0.8	41.9 ± 0.4	
3–4 days	22.4 ± 0.6	17.7 ± 0.5	13.6 ± 0.6	18.2 ± 0.4	
≥5 days	13.8 ± 0.5	12.5 ± 0.5	7.6 ± 0.4	11.7 ± 0.4	
Doing muscle strengthening exercise during the past 7 days					
None	39.8 ± 0.8	51.0 ± 0.9	65.4 ± 0.8	51.0 ± 0.8	<0.001
1–2 days	28.6 ± 0.6	26.4 ± 0.6	21.8 ± 0.7	26.0 ± 0.5	
3–4 days	16.3 ± 0.5	11.4 ± 0.4	7.6 ± 0.4	12.0 ± 0.3	
≥5 days	15.2 ± 0.5	11.3 ± 0.4	5.1 ± 0.3	11.0 ± 0.4	
Walking for ≥ 10 min during the past 7 days					
None	5.8 ± 0.3	5.6 ± 0.3	6.2 ± 0.3	5.8 ± 0.2	<0.001
1–2 days	10.2 ± 0.4	11.7 ± 0.4	12.4 ± 0.5	11.4 ± 0.3	
3–4 days	10.8 ± 0.5	12.8 ± 0.4	13.5 ± 0.5	12.4 ± 0.3	
5–6 days	18.8 ± 0.5	20.8 ± 0.5	21.8 ± 0.6	20.4 ± 0.3	
Daily	54.4 ± 0.8	49.2 ± 0.7	46.0 ± 0.7	50.0 ± 0.5	

Weighted % ± SE

### Predictors of body image overestimation

We observed that subjects from the over-estimation group were more likely to be in the 3rd year of high school, to be female, and to have lower socio-economic status compared with subjects from the correct-estimation group (Table 5). Additionally, the over-estimation group was more likely to use unhealthy weight control behaviors and to have a sadness and suicidal ideation compared with the correct-estimation group.

**Table 5:** Predictors of subjects with body image overestimation (*n* = 4,887) compared to subjects with correct body image estimation (*n* = 9,387)

Variables	Adjusted OR (95 % CI)	*P*-value
Year of high school		
1^st^ year	1.00	
2^nd^ year	1.08 (0.98, 1.19)	0.109
3^rd^ year	1.27 (1.16, 1.39)***	<0.001
Gender		
Male	1.00	
Female	3.52 (3.25, 3.81)***	<0.001
Socio-economic status		
Low	1.00	
Lower middle	1.05 (0.87, 1.26)	0.820
Middle	0.79 (0.66, 0.94)**	0.003
Upper middle	0.80 (0.66, 0.97)**	0.002
High	0.73 (0.58, 0.93)**	0.005
Unhealthy weight control behaviors		
No	1.00	
Yes	1.54 (1.37, 1.72)***	<0.001
Feeling sad or despair		
No	1.00	
Yes	1.25 (1.16, 1.35)***	<0.001
Suicidal ideation		
No	1.00	
Yes	1.20 (1.08, 1.33)**	.001

Unhealthy weight control behaviors included 1) fasting (≥24 h), 2) taking non-prescription weight loss medication, 3) taking laxatives or diuretics, 4) vomiting, 5) eating a one-food diet, and 6) eating food substitute

Statistical analysis of weighted data with adjustment for complex survey design

** *P* < 0.01, ***P < 0.001 significantly different according to the logistic regression model

### Predictors of body image underestimation

We also found that subjects from the under-estimation group were less likely to be in the higher grades of high school, to be female, and to use unhealthy weight control behaviors compared with subjects from the correct estimation group (Table 6). However, the under-estimation group was more likely to have a sadness and suicidal ideation compared with the correct estimation group.

**Table 6:** Predictors of subjects with body image underestimation (*n* = 5,990) compared to subjects with correct body image estimation (*n* = 9,387)

Variables	Adjusted OR (95 % CI)	*P*-value
Year of high school		
1^st^ year	1.00	
2^nd^ year	0.83 (0.77, 0.89)***	<0.001
3^rd^ year	0.79 (0.73, 0.86)***	<0.001
Gender		
Male	1.00	
Female	0.23 (0.21, 0.25)***	<0.001
Socio-economic status		
Low	1.00	
Lower middle	1.07 (0.88, 1.30)	0.276
Middle	0.91 (0.77, 1.09)	0.445
Upper middle	1.07 (0.88, 1.29)	0.398
High	1.07 (0.86, 1.32)	0.399
Unhealthy weight control behaviors		
No	1.00	
Yes	0.50 (0.43, 0.59)***	<0.001
Feeling sad or despair		
No	1.00	
Yes	1.12 (1.03, 1.21)*	0.026
Suicidal ideation		
No	1.00	
Yes	1.12 (1.00, 1.25)*	0.015

Unhealthy weight control behaviors included 1) fasting (≥24 h), 2) taking non-prescription weight loss medication, 3) taking laxatives or diuretics, 4) vomiting, 5) eating a one-food diet, and 6) eating food substitute

Statistical analysis of weighted data with adjustment for complex survey design

* *P* < 0.05, ***P < 0.001 significantly different according to the logistic regression model

## Discussion

The present study was aimed to investigate the prevalence of body image distortion and the factors associated with body image distortion among Korean high school students. We found that both the over-estimation and under-estimation of body weight are prevalent among normal weight high school students in South Korea. Subjects in the self-perceived overweight group were significantly more likely to be female, while the subjects of the self-perceived underweight group were significantly more likely to be male. Subjects in both the under- and over-estimation groups were significantly more likely to employ unhealthy weight control behaviors and to have a depressive mood.

This study revealed that normal weight male adolescents tend to underestimate their weight status, while normal weight female adolescents tend to overestimate their weight status. These results are similar to the previous study [16]. Mass media, peers, and parents play an important role in forming perceptions of one’s body weight status. More male adolescents received ‘increase weight’ messages, while more females received ‘lose weight’ messages from the mass media in China [16, 21, 22]. Previous studies have demonstrated that females felt greater socio-cultural pressure to adhere to a thin ideal body size and to engage in weight-loss behaviors [7, 23, 24]. On the other hand, male adolescents feel strong pressure to have a more muscular figure [22]. Among boys, male peers were especially influential in supporting muscle building practices [25, 26], while among girls, female peers were influential in promoting weight loss [26]. Parental influence has also been demonstrated to impact the formation of an ideal body type in children by promoting specific dietary habits and exercises, and also by displaying their own habits related to diet and physical activity and talking about their body image concerns [27]. This suggests that we need to educate adolescents to critically evaluate mass-media images and messages. Additionally, it would be important to include parents and peer group education due to their strong influence on the body image perception of adolescents.

Practices of unhealthy behaviors are more common in the over-estimation group and the under-estimation group compared to the correct estimation group. The over-estimation group performed more unhealthy weight control behaviors such as fasting and eating one food diet, the easy and quick ways to control their weight in a short period of time. However, these practices could increase the risk of anemia, osteoporosis, and dehydration [4] and also cause clinical eating disorders [5]. The under-estimation group consume high calorie, low nutrient foods such as fast-food and soda relatively more frequently than the other two groups. Consequently, we need to develop targeted nutrition messages for the over-estimation and the under-estimation group respectively.

Both self-perceived underweight and overweight subjects were more likely to have feeling sad or despair and suicidal ideation compared to subjects who correctly estimated their body weight status. As previously described, male adolescents tend to feel pressure to have a muscular body, while female adolescents tend to feel pressured to have a thin body. Consequently, a discrepancy between current and ideal BMI exists and can lead to feeling sad or despair and suicidal ideation [28]. Adolescents with body image distortion have felt they failed to achieve an ideal body and have lower self-esteem in spite of their normal body weight [28]. Additionally, adolescents’ body weight misperceptions could lead to bullying by peers because low self-esteem has been identified as the target of bullying behavior [29]. This hypothesis is supported by a study that found a U-shaped relationship between body image and exposure to bullying. Brixval *et al*. reported that both males’ under-estimations and females’ over-estimations of their own weight were related to higher risks of bullying [30]. Therefore, these findings suggest that nutritional interventions are needed to help adolescents adapt correct body images in order to preserve their emotional health.

This study has several major strengths. First, we used nationally representative data to assess the degree of body weight misperception at the national level. Second, we used a comprehensive approach to identify associations between body image distortion and various related factors (i.e., weight control behaviors, dietary behaviors, and physical activity). Third, we would produce a number of predictors of self-perceived underweight and overweight subjects compared with students who perceived their body weight accurately among normal weight adolescents, acknowledging the use of categorical data and its limitations in this study. This study is critically important because most studies have focused on body weight overestimation but not body weight underestimation.

There are a few limitations to this study. First, due to its cross-sectional nature, we could examine associations between risk factors (i.e., weight control behaviors, sadness, and suicidal ideation) and body image distortion. However, we cannot determine their causal relationships. Second, self-reported height and weight data are not as accurate as directly measured height and weight. Third, the frequency of food (or food group) consumption gives a general idea of diet quality. However, this method could not provide accurate measures of the amounts of food eaten and nutritional intake. To overcome the limitations of this study, future studies should use the 24-h diet recall method to investigate the food and nutrient intake level by body image perception. Fourth, we used self-report questionnaires to obtain data such as weight control attempts and behaviors, dietary behaviors, and physical activity. Although self-report designs are the easy and quick ways to collect data, this data would be subjective to social desirability. Some sensitive questionnaires such as mental health items could be under-reported due to stigma. Lastly, sadness and suicidal ideation were measured by single measure item/question instead of comprehensive instruments.

This empirical research has several policy and program implications. First, in South Korea, nutrition intervention programs have focused on weight-loss programs targeting obese adolescents. However, we found that it is also imperative to monitor the subjective perception of body weight among adolescents. Body image distortion, both the under-estimation and over-estimation of body weight, should no longer be neglected because it is a critical determinant of mental health and weight control behaviors. Second, we urgently need to develop a gender-specific nutrition intervention program aimed at reducing body image distortion, including the over-estimation of one’s own body weight among female adolescents and the under-estimation of one’s own body weight among male adolescents. The findings of this study will contribute to understanding the factors associated with body image distortion among high school students in South Korea.

## Conclusions

The aim of the present study was to investigate the prevalence of body image distortion and the factors associated with body image distortion among Korean high school students. Both the over-estimation and under-estimation of body weight were prevalent among normal weight high school students in South Korea. These findings showed that normal weight female students tend to overestimate their own body weight, while normal weight male students tend to underestimate their own body weight. We found that subjects in the self-perceived overweight group were significantly more likely to be older and female, to have lower socio-economic status and to employ unhealthy weight control behaviors compared with the correct body weight estimation group. On the other hand, we found that the subjects of the self-perceived underweight group were significantly more likely to be younger and male compared with the correct body weight estimation group. Subjects in both the under- and over-estimation groups were significantly more likely to have a sadness and suicidal ideation. This study provides critical information for developing nutrition policy and nutrition intervention programs in South Korea to reduce the prevalence of distorted body image by understanding how demographic factors, weight control behaviors, and mental health characteristics are associated with perceived body weight distortion.

## Additional file

Additional file 1: Table S1. International child cut-offs corresponding to BMI cut-offs at 18 years. **Table S2.** Agreement between body image and actual body weight status based on the BMI of high school students, by gender. (DOCX 19 kb)

## Abbreviations

AOR: adjusted odds ratio
CI: confidence interval
BMI: body mass index
