# Supplementary material for: The association of body image distortion with weight control behaviors, diet behaviors, physical activity, sadness, and suicidal ideation among Korean high school students: a cross-sectional study
Source: BMC Public Health. 2016 Jan 15;16:39. doi: 10.1186/s12889-016-2703-z (PMC4714421; doi:10.1186/s12889-016-2703-z)
Supplement: Additional file 1: Table S1. — International child cut-offs corresponding to BMI cut-offs at 18 years. Table S2. Agreement between body image and actual body weight status based on the BMI of high school students, by gender. (DOCX 19 kb) [file 12889_2016_2703_MOESM1_ESM.docx]

**Table S1.** International child cut-offs corresponding to BMI cut-offs at 18 years

|  | BMI (kg/m^2^) at age 18 years | | | |
| --- | --- | --- | --- | --- |
| Age (years) | Male | Female | Male | Female |
|  | 18.50 | 18.50 | 23.00 | 23.00 |
| 15 | 16.98 | 17.43 | 21.31 | 21.88 |
| 16 | 17.53 | 17.90 | 21.92 | 22.35 |
| 17 | 18.04 | 18.24 | 22.48 | 22.60 |
|  |  |  |  |  |

BMI, body mass index

**Table S2.** Agreement between body image and actual body weight status based on the BMI of high school students, by gender

|  | Based on BMI | | |  | |  |
| --- | --- | --- | --- | --- | --- | --- |
| Subjective body image | Underweight  (n=3,550) | Normal weight  (n=20,264) | Overweight or obese  (n=10,946) | Overall agreement  (%) | Kappa  (95%CI) | |
| All |  |  |  |  |  | |
| Thin | 2,979 (8.57) | 5,990 (17.23) | 93 (0.27) | 61.48 | 0.41 | |
| Normal | 477 (1.37) | 9,387 (27.01) | 1,851 (5.33) |  | (0.40, 0.42) | |
| Fat | 94 (0.27) | 4,887 (14.06) | 9,002 (25.90) |  |  | |
| Males |  |  |  |  |  | |
| Thin | 1,327 (7.66) | 4,563 (26.32) | 89 (0.51) | 58.83 | 0.39 | |
| Normal | 72 (0.42) | 3,869 (22.32) | 1,620 (9.35) |  | (0.38, 0.40) | |
| Fat | 14 (0.08) | 779 (4.49) | 5,001 (28.85) |  |  | |
| Females |  |  |  |  |  | |
| Thin | 1,652 (9.48) | 1,427 (8.19) | 4 (0.02) | 64.11 | 0.44 | |
| Normal | 405 (2.32) | 5,518 (31.67) | 231 (1.33) |  | (0.43, 0.45) | |
| Fat | 80 (0.46) | 4,108 (23.57) | 4,001 (22.96) |  |  | |

BMI, body mass index; CI, confidence interval
